# Schwannoma of the Median Nerve at the Wrist and Palmar Regions of the Hand: A Rare Case Report

**DOI:** 10.1155/2013/950106

**Published:** 2013-08-24

**Authors:** Harun Kütahya, Ali Güleç, Yunus Güzel, Burkay Kacira, Serdar Toker

**Affiliations:** ^1^Department of Orthopaedics and Traumatology, Konya Beyhekim State Hospital, 42100 Selçuklu, Konya, Turkey; ^2^Department of Orthopaedics and Traumatology, Konya Medical Training and Research Hospital, Konya, Turkey; ^3^Yozgat Akdağmadeni State Hospital, Yozgat, Turkey; ^4^Department of Orthopaedics and Traumatology, NE University, Meram School of Medicine, Konya, Turkey; ^5^Division of Hand and Upper Extremity Surgery, Department of Orthopaedics and Traumatology, NE University, Meram School of Medicine, Konya, Turkey

## Abstract

Schwannomas are also known as neurolemmas that are usually originated from Schwann cells located in the peripheric nerve sheaths. They are the most common tumours of the hand (0.8–2%). They usually present solitary swelling along the course of the nerve however multiple lesions may be present in cases of NF type 1, familial neurofibromatosis, and sporadic schwannomatosis. Schwannomas are generally represented as an asymptomatic mass; however pain, numbness and fatigue may take place with the increasing size of the tumour. EMG (electromyelography), MRI (magnetic resonance imagination), and USG (ultrasound) are helpful in the diagnosis. Surgical removal is usually curative. In this paper, we present a 24-year-old male referred to our clinic for a lump located at the volar side of the left wrist and a lump located in his left palm and numbness at his 3rd and 4th fingers. Total excision was performed for both lesions. Histopathological examination of the masses revealed typical features of schwannoma. At the 6th-month followup the patient was symptom-free except for slight paresthesia of the 3rd and the 4th fingers. For our knowledge, this is the second case in the literature presenting wrist and palm involvement of the median nerve schwannoma.

## 1. Introduction

Benign tumours involving peripheral nerves of the upper extremity are uncommon [1] and they cause difficulties in classification, clinical diagnosis, and treatment. In the upper limb, they may be mistaken for ganglia or carpal tunnel syndrome [2]. Schwannomas also known as neurolemmas are usually originated from Schwann cells located in the peripheric nerve sheats. They are the most common tumours of the hand (0.8–2%) [3]. They usually grow slowly and appear as painless swellings for several years before diagnosed [4]. They usually present with solitary swelling along the course of the nerve [1], however multiple lesions may be present in cases of NF type 1, familial neurofibromatosis, and sporadic schwannomatosis. Incidence is similar between both genders and the tumour is the most common in 3rd and 6th decades. Schwannomas are generally represented as an asymptomatic mass; however pain, numbness, and fatigue may take place with the increasing sizes of the tumour; however it is unusual for a schwannoma to exceed three centimeters in diameter [1]. EMG, MRI, and USG are helpful in the diagnosis. Surgical removal is usually curative.

## 2. Case Report

**Figure 1:**
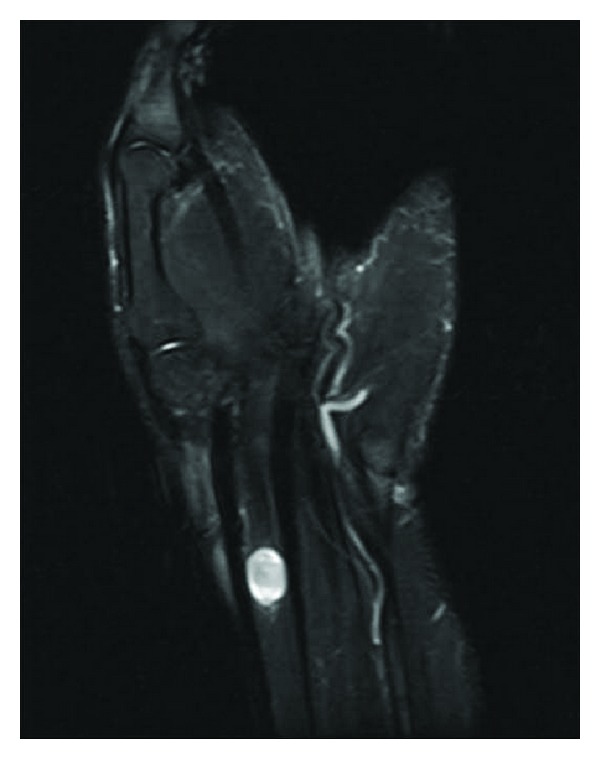
MRI revealed a 11 × 9 mm mass located in flexor tendons which has intermediate signal on T1-weighted images and hyperintense signal on T2-fat weighted images.

**Figure 2:**
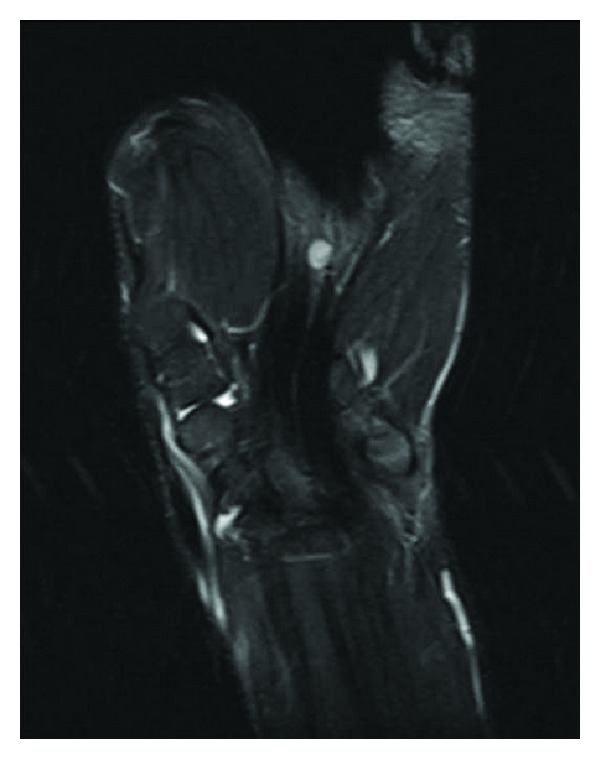
6 mm diameter mass in the palm which has intermediate signal on T1-weighted images and hyperintense signal on T2-fat weighted images was detected.

A 24-year-old male was referred to our clinic for a lump located at the volar side of the left wrist and a lump located in his left palm and numbness at his 3rd and 4th fingers. In his history he was aware of the masses for 2 years but the numbness was only present for the last 3 months. Lumps were diagnosed as ganglias in another clinic. In physical examination a 2 × 2 cm lump at the volar side of the wrist (zone 5) and 0.5 × 0.5 cm lump at the level of palmar crisis were detected. Numbness was spotted on the ulnar side of the 3rd finger and on the radial side of the 4th finger. The lumps were nontender with no radiating pain. A positive Tinel sign at the wrist and palm and a Phalen sign were spotted. There was no objective motor deficit. There was no family history of neurofibromatosis and no associated clinical features. USG revealed a 12 × 14 mm mass at the volar side of the wrist and 4 × 5 mm mass in the palm which both were in close relation with flexor tendons and had no blood supplies. MRI revealed a 11 × 9 mm mass located in flexor tendons which has intermediate signal on T1-weighted images and hyperintense signal on T2-fat weighted images (Figure 1). Also a second 6 mm diameter mass in the palm with the same properties was detected (Figure 2). The masses were both encapsulated with remarkable borders.

**Figure 3:**
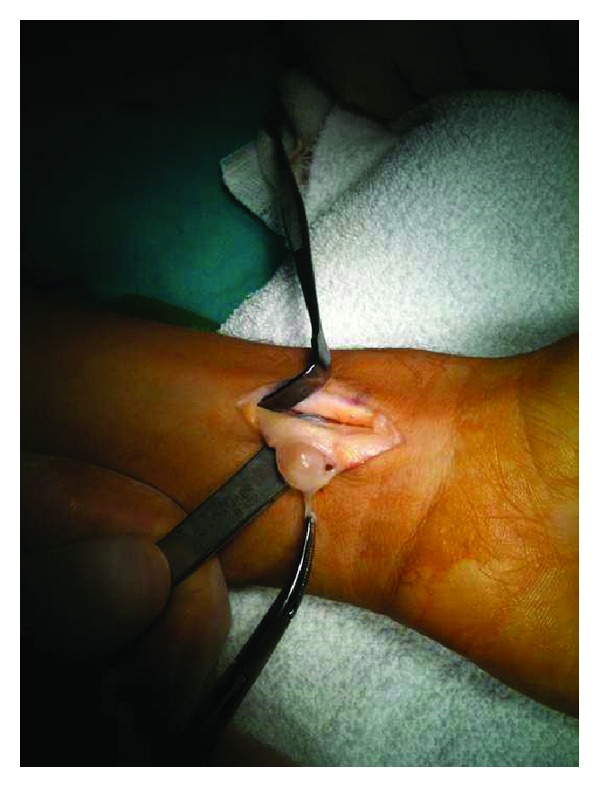
Intraoperative view of the lesion showing that the mass at the wrist was originated from the median nerve.

**Figure 4:**
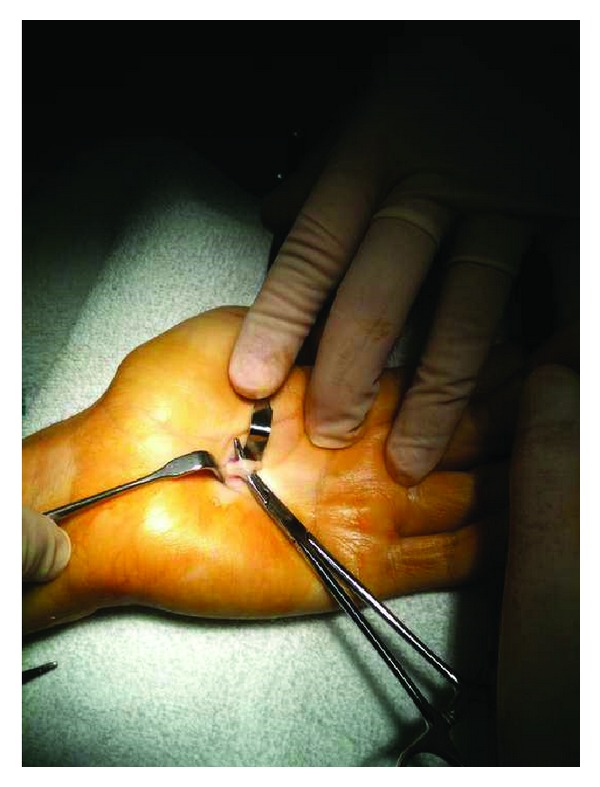
Intraoperative view of the lesion showing that the mass in the palm was originated from the common digital nerve of the 3rd and the 4th fingers.

Total excision  was performed for both lesions. The mass at the wrist was originated from the median nerve (Figure 3) and the mass in the palm was originated from the common digital nerve of the 3rd and the 4th fingers (Figure 4). Microsurgical techniques were used to resect the 15 × 15 mm mass from the median nerve. The palm was opened with a separate incision and a smaller mass with a 6 mm diameter was also removed from the common digital nerve. The masses were encapsulated and removed totally. Histopathological examination of the masses revealed typical features of schwannoma. At the 6th-month followup the patient was symptom-free except slight paresthesia of the 3rd and the 4th fingers. There were no motor deficit or pain and no recurrence of the lumps.

## 3. Discussion

Schwannomas are rare tumours [5]. They are usually solitary and benign lesions; however they can be multiple suggesting an underlying tumour predisposition syndrome [6] and may be associated with neurofibromatosis type 1 and schwannomatosis [7]. In the last 15 years, there have been several reports of multiple schwannomas with no evidence of a vestibular tumour leading schwannomatosis being considered a clinical entity different from other forms of neurofibromatosis [6]. The incidence of multiple schwannomas has been reported as 1% to 23%. The mostly affected nerves are ulnar and median nerves [8]. There are several cases of multiple schwannomas of median nerve reported in the literature [9–14]. Wrist and palm involvement similar to our case is reported only in one of these studies [15].

The reported interval between onset of symptoms and surgery has varied from a few months to years. [4] In our patient the interval between clinical appearance and symptoms was two years and surgery was performed three months after the symptoms.

These tumours are slow growing, soft in consistency, mobile in nature, and sometimes painless so they may be misdiagnosed as lipoma, fibroma, ganglion, or xanthoma. Holdsworth [16], White [17], and Phalen [10] reported low rates of correct diagnosis. The lesions in our patient were also misdiagnosed as ganglia in another clinic. Also, we were not sure about the diagnosis at the first examination so we needed to confirm it with USG and MRI. However both examinations were not successful in diagnosing schwannomas. MRI can provide useful information about morphological data on the median nerve tumours; however it cannot provide dynamic information [18]. Although low intense signals on TI-weighted images and hyperintense signals on T2-weighted images are common findings of schwannomas, lesions were reported as complicated cystic lesions or giant cell tumour of tendon sheath in MRI at our case. Conversely, USG gives detailed informative images during static and dynamic positions such as flexion and extension maneuvers, showing the nerve in relation to the surrounding musculotendinous structures [19]. We did not perform dynamic examination and USG only revealed cystic lesions without any blood supply.

Surgical excision is the most effective method of theraphy [3, 10]; however some authors recommend excision of only symptomatic tumours or those demonstrating enlargement during followup [6, 20]. Careful microsurgical dissection in a bloodless field is important [2] so the use of loupe or microscopical magnification are advised to avoid damaging the nerve fibers during the epineural and endoneurial dissection. Paresthesia is the most common postoperative complication. [21] The surgeon must be careful not to make unnecessary sacrifice of functionally important motor and sensory branches. In this case, we also used microsurgical technique to remove the tumour and tried to protect the nerve; however a slight paresthesia is consisting on at the 3rd and the 4th fingers.

## Supplementary Material

Figure 1: MRI revealed a 11×9 mm mass located in flexor tendons which has intermediate signal on T1-weighted images and hyperintense signal on T2-fat weighted images. Figure 2: 6 mm diameter mass in the palm which has intermediate signal on T1-weighted images and hyperintense signal on T2-fat weighted images was detected. Figure 3: Intraoperative view of the lesion showing that the mass at the wrist was originated from the median nerve. Figure 4: Intraoperative view of the lesion showing that the mass in the palm was originated from the common digital nerve of the 3rd and the 4th fingers.
