# Supplementary material for: Schwannoma of the Median Nerve at the Wrist and Palmar Regions of the Hand: A Rare Case Report
Source: Case Rep Orthop. 2013 Aug 24;2013:950106. doi: 10.1155/2013/950106 (PMC3766574; doi:10.1155/2013/950106)
Supplement: Supplementary file 1 — Figure 1: MRI revealed a 11×9 mm mass located in flexor tendons which has intermediate signal on T1-weighted images and hyperintense signal on T2-fat weighted images. Figure 2: 6 mm diameter mass in the palm which has intermediate signal on T1-weighted images and hyperintense signal on T2-fat weighted images was detected. Figure 3: Intraoperative view of the lesion showing that the mass at the wrist was originated from the median nerve. Figure 4: Intraoperative view of the lesion showing that the mass in the palm was originated from the common digital nerve of the 3rd and the 4th fingers. [file 950106.f1.docx]

FIGURES


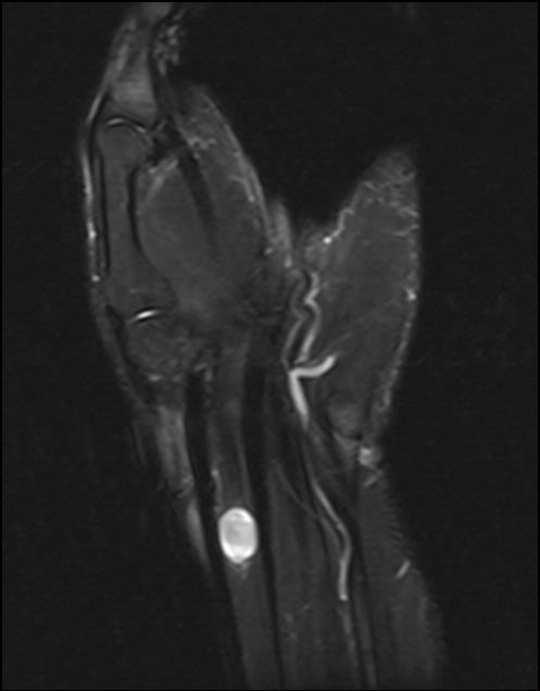


**Figure 1:** MRI revealed a 11x9 mm mass located in flexor tendons which has intermediate

signal on T1-weighted images and hyperintense signal on T2-fat weighted images.


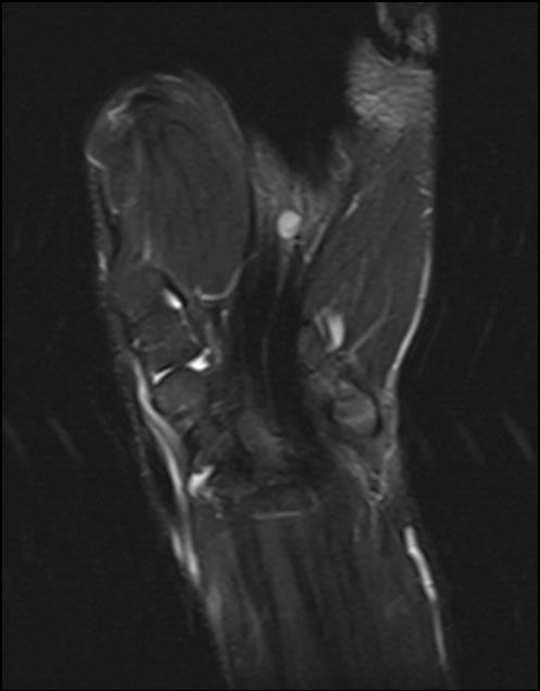


**Figure 2:** 6 mm diameter mass in the palm which has intermediate signal on T1-weighted

images and hyperintense signal on T2-fat weighted images was detected.


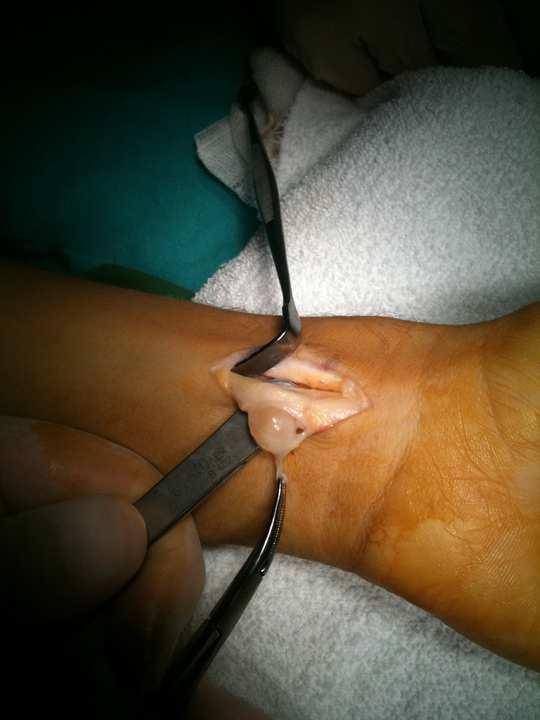


**Figure 3:** Intraoperative view of the lesion showing that the mass at the wrist was originated

from the median nerve.


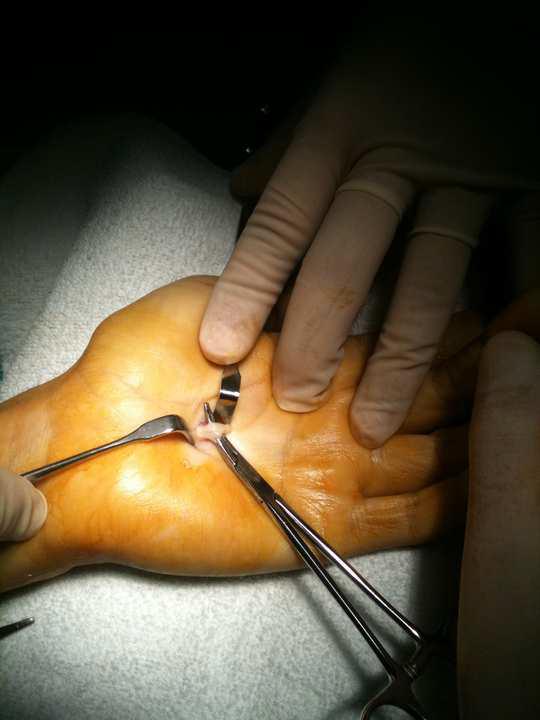


**Figure 4:** Intraoperative view of the lesion showing that the mass in the palm was originated

from the common digital nerve of the 3^rd^ and the 4^th^ fingers.
